# Case report: Hepatic tuberculosis mimicking hepatocellular carcinoma in a patient with cirrhosis induced by hepatitis B virus

**DOI:** 10.3389/fmed.2022.1005680

**Published:** 2022-11-15

**Authors:** Na Hu, Yuhui Wu, Maowen Tang, Tianyong Luo, Shengmei Yuan, Cai Li, Pinggui Lei

**Affiliations:** ^1^Department of Radiology, Affiliated Hospital of Guizhou Medical University, Guiyang, China; ^2^Department of Infection, Affiliated Hospital of Guizhou Medical University, Guiyang, China; ^3^Department of Ultrasound Center, Affiliated Hospital of Guizhou Medical University, Guiyang, China

**Keywords:** hepatic tuberculosis, portal vein thrombosis, hepatitis B virus, cirrhosis, hepatocellular carcinoma, case report

## Abstract

Hepatic tuberculosis (TB), which is secondary to post-hepatitis B cirrhosis, is extremely rare. We report the case of a 69-year-old man with post-hepatitis B cirrhosis complicated by primary isolated hepatic TB who was initially misdiagnosed as having hepatocellular carcinoma (HCC). The patient was hospitalized with complaints of 2 weeks of fever. He had a 20-year history of post-hepatitis B cirrhosis. The laboratory tests suggested that his serum alpha-fetoprotein (AFP) level was markedly elevated to 1210 ng/ml. From the abdominal ultrasound (US) and magnetic resonance imaging (MRI) images, we confirmed the presence of cirrhosis and discovered a space-occupying lesion of the hepatic left lobe as well as portal vein-filling defects. These results led us to consider primary liver cancer and portal vein tumor thrombus combined with decompensated cirrhosis. Biopsy and histology may be considered the ultimate diagnostic tests, but we excluded needle biopsy because of his high risk of bleeding, in addition, the patient declined the procedure. To cope with his fever, the patient was given broad-spectrum antibiotic treatment initially, followed by intravenous vancomycin. After antibiotic treatment had failed, the patient was treated with anti-TB for 10 days; after that, the patient maintained a normal temperature. The patient continued to receive tuberculostatic therapy for 6 months following his discharge. AFP completely returned to the normal level, and the aforementioned mass disappeared. Finally, hepatic TB secondary to post-hepatitis B cirrhosis with portal vein thrombosis (PVT) was considered to be the final diagnosis. More than two imaging techniques discover a space-occupying liver lesion and that the serum alpha-fetoprotein (AFP) level is extremely elevated, which means that hepatocellular carcinoma (HCC) could be diagnosed. However, some exceedingly rare diseases should not be excluded. This case illustrated that the non-invasive diagnostic criteria for liver cancer should be considered carefully when discovering a space-occupying liver lesion in a patient with cirrhosis and an elevated AFP level. In addition, primary hepatic TB should be considered and included in the differential diagnosis, and a biopsy should be performed promptly.

## Introduction

Primary hepatic tuberculosis (TB) is rare and considered to occur without pulmonary TB. Clinical manifestations and radiological characteristics are not specific, leading to difficulties in diagnosis. Imaging is an important factor in recognizing patients who need an invasive examination or potential treatment in the differential diagnosis between benign and malignant liver lesions. If a space-occupying liver lesion is discovered in more than two imaging techniques and the serum AFP level in the peripheral blood is extremely elevated, then hepatocellular carcinoma (HCC) diagnosis could be confirmed. However, some exceedingly rare diseases should not be excluded. In our study, we described the case of a 69-year-old man with a history of post-hepatitis B cirrhosis who presented with a hepatic space-occupying lesion simultaneously with an extremely elevated AFP level and was finally diagnosed with primary hepatic TB.

## Case presentation

**Figure 1:**
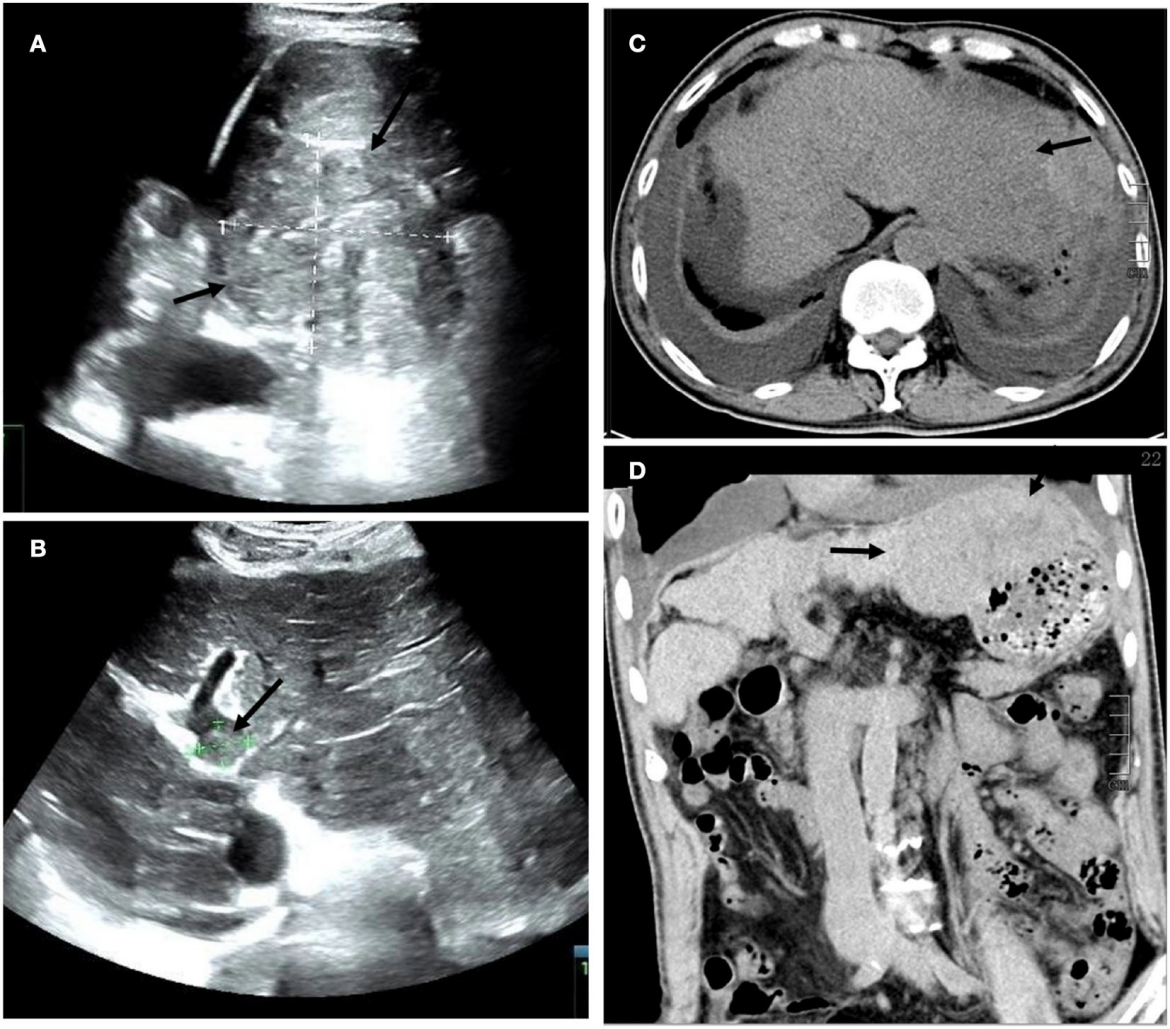
**(A)** Transverse ultrasound (US) images of a large hypoechoic lesion with an irregular border and non-uniform internal echoes in the left lobe of the liver. Additionally **(B)** there is a filling defect approximately 1.7 cm in diameter within the trunk of the portal vein (arrow). **(C)** Axial and **(D)** coronal plain abdominal CT scans revealed a decompensated period of cirrhosis, a lobulated and inhomogeneous mass with a slightly lower density in the left lobe, measuring 13.5 cm x 6.8 cm, and the presence of ascites and bilateral pleural effusion (arrow).

**Figure 2:**
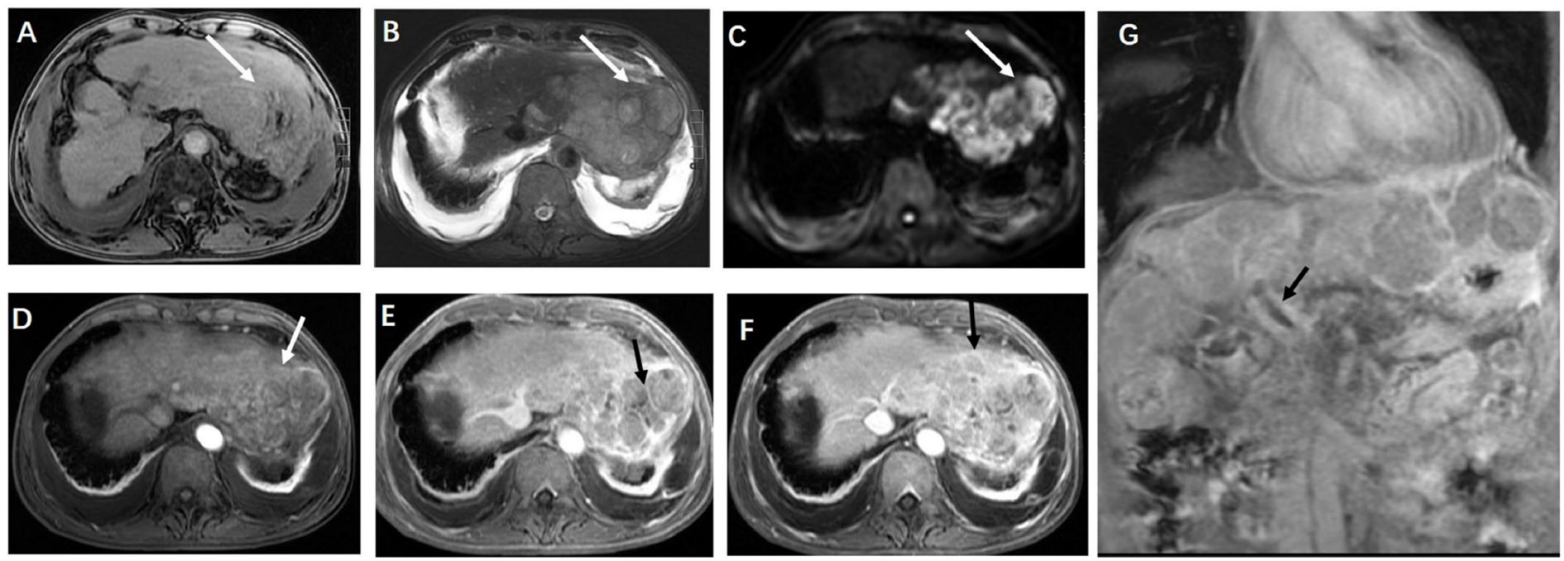
**(A)** Axial T1-weighted magnetic resonance imaging (MRI) and **(B)** axial T2-weighted MRI scans showed a bulky multicystic hepatic mass with multiple septa (arrowheads) situated in the left hepatic external lobe, and **(C)** a diffusion-weighted MRI scan presented a restriction of diffusion. Axial contrast-enhanced T1-weighted MRI scans showed local enhancement in the lesion, and most of the lesion suggested the absence of being not significantly obviously enhanced in the arterial phase **(D)**, and mild to moderate delayed enhancement was found in the perifocal and internal septum of the portal venous phase **(E)** and the delayed phase **(F)** (arrow) **(G)** Coronal contrast-enhanced presented with a filling defect in the trunk of the portal vein (arrow).

A 69-year-old man was diagnosed with post-hepatitis B cirrhosis for 20 years and presented with 2 weeks of fever. Two weeks before his admission, he developed pyrexia without obvious predisposing causes, and his highest temperature recorded was 39.6°C, with fever ocurring mainly in the afternoons. Furthermore, the patient suffered from asthenia, anorexia, mild cough, whitish sputum, and pain in the anterior chest wall without chills or abdominal pain. He had undergone a splenectomy for hypersplenism 16 years ago. During a physical examination, his temperature was 38.8°C. There were many spider angiomas in the anterior thoracic wall and decreased respiratory sound in both lungs, combined with shifting dullness and fist percussion over the liver. Laboratory tests revealed a C-reactive protein level of 84.36 mg/L (normal range <5 mg/L) and an erythrocyte sedimentation rate (ESR) level of 69 mm/h (normal range 0–43 mm/h). Liver function tests demonstrated a slight elevation of aspartate aminotransferase (AST) at 59.7 U/L, total bilirubin (TBIL) at 39.7 mmol/L, and a slight decrease of albumin at 31.4 g/L. Additionally, serum alpha-fetoprotein (AFP) level was markedly elevated to 1210 ng/ml (normal range 0–20 ng/mL). The serum adenosine deaminase (ADA) was mildly elevated to 24.4 U/L. However, an ascitic fluid examination demonstrated that ADA was normal. Negative test results were observed for purified protein derivative (PPD) and mycobacterium TB DNA. An interferon-gamma release assay (IGRA) test was positive. The results of coagulation function tests expressed that prothrombin time (PT) was 19.9 s (normal range 10–15 s), the international normalized ratio (INR) was 1.71, and there was 47.7% prothrombin time activity (PTA). An ultrasound (US) of the abdomen revealed signs of cirrhosis, a filling defect in the portal vein, and a large hypoechoic lesion with an irregular border and non-uniform internal echoes in the left lobe of the liver. Abdominal CT plain scans depicted a decompensated period of cirrhosis, a lobulated and inhomogeneous mass with a slightly lower density in the left lobe, measuring 13.5 cm x 6.8 cm, and the presence of ascites and bilateral pleural effusion (Figure 1). MRI, including T1- and T2-weighted imaging and contrast-enhanced with fat saturation and subsequent subtraction, revealed signs of cirrhosis and a huge mass in the left liver lobe, characterized by slightly higher non-uniform intensity, which was not significantly enhanced in the arterial phase, and mild to moderate delayed enhancement was found in the perifocal and internal septum of the venous phase and the delayed phase. In addition, the mass presented without a capsule surrounding it. The mass was hyperintense in DWI mapping. Moreover, a filling defect in the trunk of the portal vein was observed (Figure 2). The results led us to consider primary liver cancer and main portal vein tumor thrombus combined with decompensated cirrhosis.

Although discovering a space-occupying lesion of the liver on MRI and that the AFP level in the peripheral blood was extremely elevated, the first diagnosis was probably HCC. Biopsy and histology may be considered the ultimate diagnostic tests, but needle biopsy was excluded because of a high risk of bleeding; in addition, the patient declined the procedure.

To cope with fever, the patient was initially given broad-spectrum antibiotic treatment (3.0 g of cefoperazone-sulbactam every 8 h for 6 days and 0.5 g of levofloxacin every 24 h for 3 days, followed by 0.5 g of intravenous vancomycin every 12 h and 1 g of meropenem every 8 h *via* intravenous drip infusion for 3 days). After 12 days, the fever persisted (the highest temperature recorded was 38.2°C.). Although there was no direct evidence of TB infection, primary hepatic TB was suspected based on clinical symptoms, and an anti-TB therapeutic diagnosis was given. The patient was treated with 0.75 g of anti-TB oral ethambutol every 24 h and 0.6 g of amikacin every 24 h *via* intravenous drip infusion for 10 days; after that, the patient maintained a normal temperature (36.5°C). Anti-TB treatment continued at the same dosage for the following 15 days, with supplemental isoniazid at 0.3 g/24 h. In the days and weeks following the patient's coagulopathy correction, we repeatedly offered him a biopsy, but he declined it. The patient was then discharged from the hospital.

**Figure 3:**
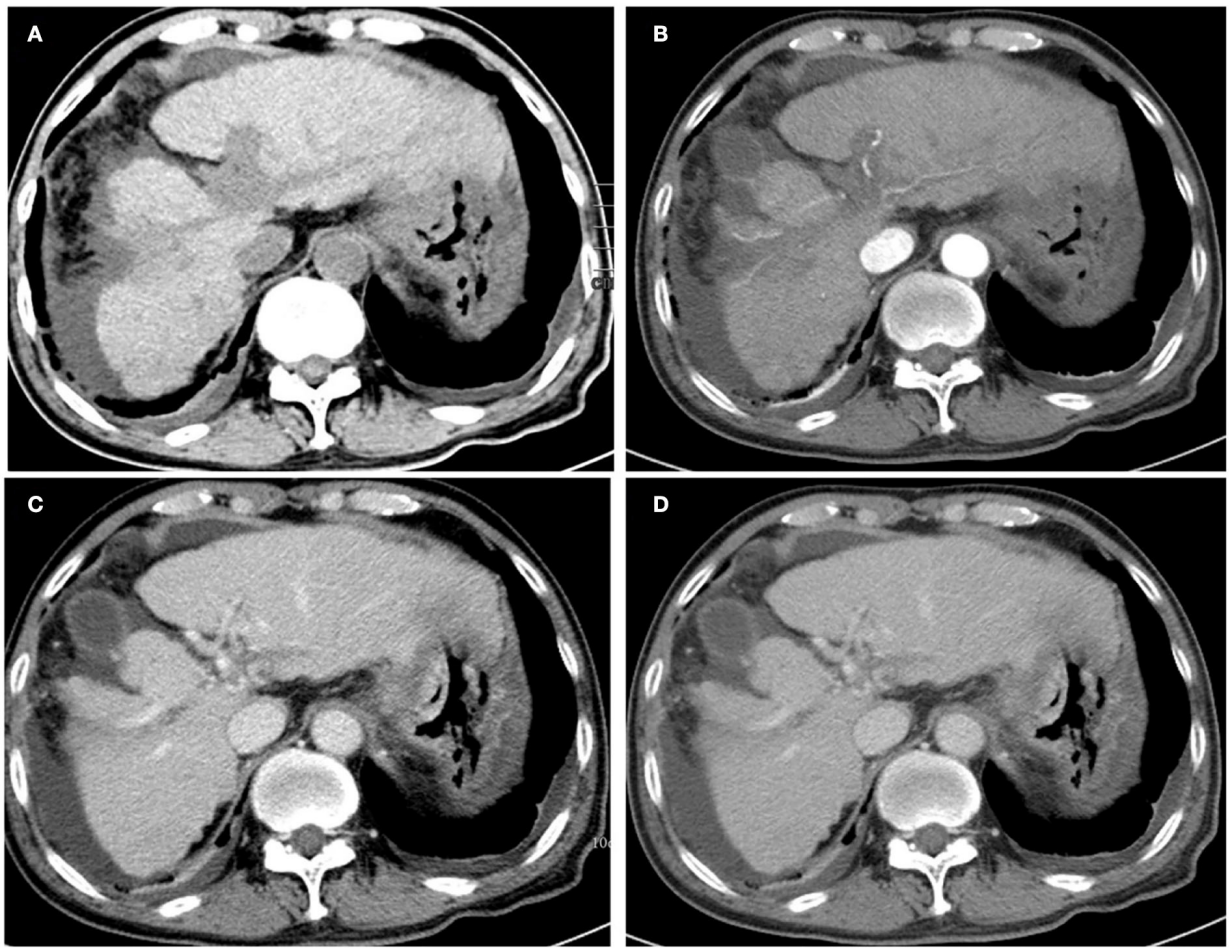
Unenhanced **(A)** and contrast-enhanced abdominal CT images were obtained 20 weeks after the initial presentation. The mass on the left liver lobe disappeared, and the portal vein-filling defect was markedly reduced after post-therapy imaging. **(B)** The axial arterial phase. **(C)** The axial portal venous phase, and **(D)** the delay phase. Note that the bilateral pleural effusion indicated significant absorption.

**Figure 4:**
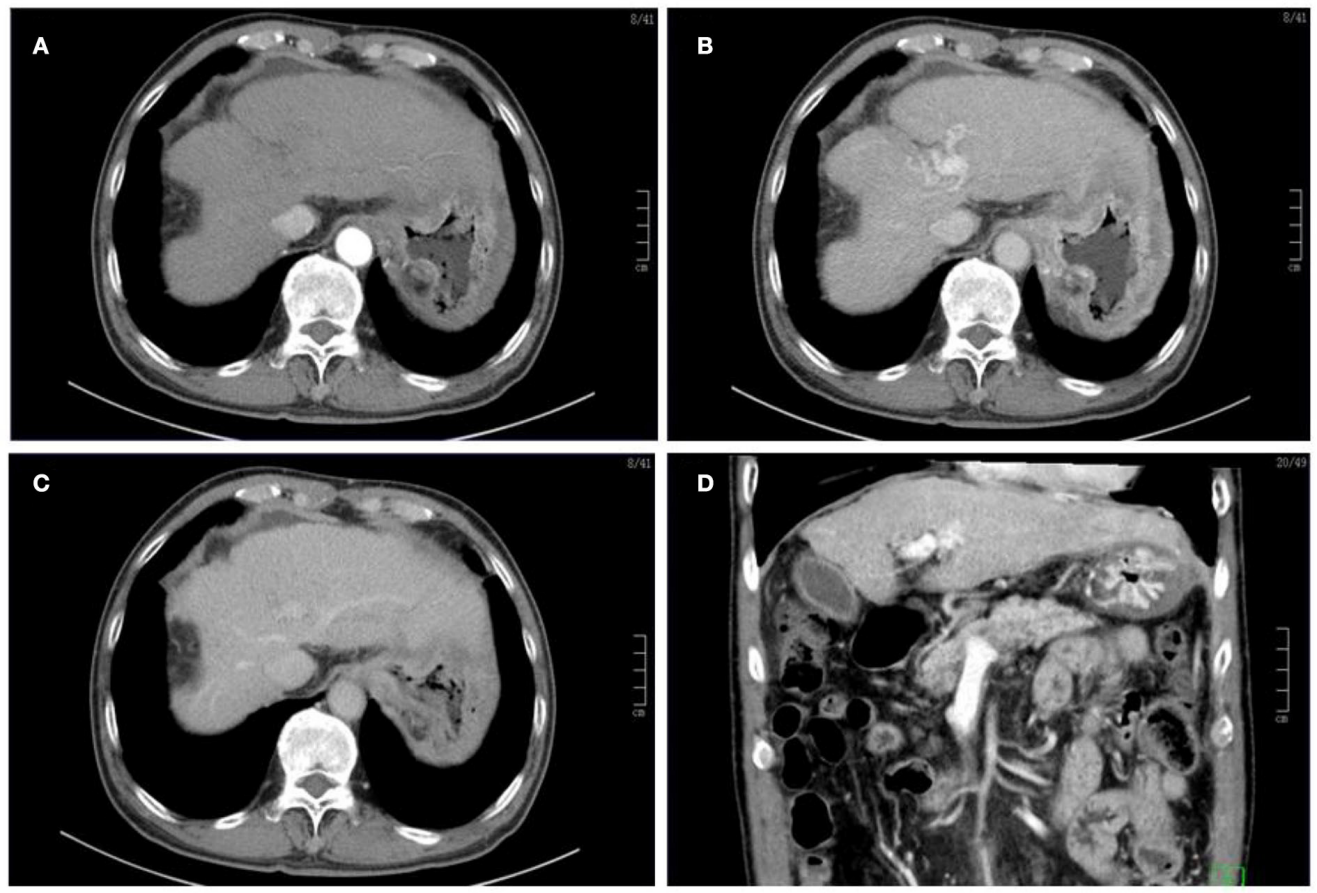
Following the initial presentation, enhanced CT was obtained 2 years later, which revealed that the mass on the left liver lobe and the portal vein-filling defect had disappeared. Note that the intraperitoneal effusion had been also absorbed **(A–D)**.

The patient received tuberculostatic therapy with amikacin, isoniazid, and ethambutol for 6 months. The level of AFP had completely normalized upon review after stopping the medication for 20 weeks, and the imaging displayed that the mass on the left liver lobe had disappeared, the portal vein filling defect had markedly reduced, and ascites and bilateral pleural effusion reduced in the patient (Figure 3). At the 2-year follow-up, no evidence of recurrence was observed (Figure 4), and the patient had recovered well. The final diagnosis was predominantly that of hepatic TB based on the disappearance of the lesions, and the level of AFP gradually returned to normal after the patient had been given anti-TB therapy for 6 months.

## Discussion

The patient who presented with recurrent fever was referred to our institution with a high suspicion of HCC because of his history of hepatitis cirrhosis and his markedly elevated level of AFP. However, there are multiple reasons for the cause of pyrexia, mainly distinguishing between infectious vs. non-infectious etiology.

In this case, the patient had a 20-year history of post-hepatitis B cirrhosis; therefore, discriminating between infectious and neoplastic fever upon imaging and laboratory testing is significant. More than two imaging techniques discovered a space-occupying lesion of the liver and that the serum AFP level in peripheral blood is extremely elevated, which means that HCC could be diagnosed, but laboratory tests indicated that infectious fever, such as, *Mycobacterium tuberculosis* infections, could not be excluded due to his clinical presentation of afternoon fever and being IGRA-positive, along with his levels of ESR and ADH being elevated.

Imaging is an important factor in recognizing patients who need an invasive examination or potential treatment in the differential diagnosis between benign and malignant liver lesions. In this case, malignant lesions, such as HCC and intrahepatic cholangiocarcinoma (ICC), and metastases should be considered. On the spectrum of benign diseases, the leading differential diagnosis would be that of a pyogenic hepatic abscess.

At first, for a space-occupying lesion of the liver against the backdrop of hepatic cirrhosis, the primary consideration would be HCC. However, contrast-enhanced CT or MRI findings in HCC have their own hallmark imaging reinforcement characteristics. Unlike, in this case, the obvious enhancement is included in the early phase, and such enhancement declined in the venous phase, which is very typical for this diagnosis. Nevertheless, the level of AFP in the patient in peripheral blood was extremely elevated, which can mislead to our diagnosis. Notwithstanding, the AFP level correlate with the regenerative capacity of the hepatoblasts and therefore was found to be increased after liver injury, thus, benign liver diseases, such as, liver cirrhosis and chronic hepatitis, have an elevated serum AFP level (1, 2).

Intrahepatic cholangiocarcinoma could also be considered, as it also has a multiloculated cystic appearance. Nevertheless, the clinical presentation of cholangiocellular carcinoma would probably be that of obstructive jaundice, and the tumor marker of CA199 might also be elevated. In addition, the imaging features of ICC manifested as a progressive contrast enhancement during the arterial and portal phases and the delayed phase depicted that the intrahepatic biliary duct was dilated.

Furthermore, metastasis should be considered a potential differential diagnosis. Nonetheless, it was rapidly excluded due to the clinical history of the patient. He had a history of a primary tumor or imaging examination discovered upon another primary tumor, especially mucinous adenocarcinoma of the gastrointestinal. In this case, the other body systems examinated by CT were oncologically negative. Upon CT or MRI, multiple liver metastases would appear and typically present as being peripherally enhanced.

On the spectrum of benign diseases, the leading differential diagnosis would be that of a pyogenic hepatic abscess. The clinical presentation of a pyogenic hepatic abscess also included fever, but it would probably be more acute and present with high-grade fever associated with chills. Unlike this case, the results of a white blood cell count would be significantly elevated. Upon MRI or CT, the abscess wall displayed obvious reinforcement, and the internal fluid area was unenhanced. Sometimes the gas-liquid level was visible, and the abscess cavity would probably show high signal intensity on diffusion-weighted images (DWI) with a low apparent diffusion coefficient (ADC) value.

The final diagnosis was predominantly that of hepatic TB based on the disappearance of the lesions, and the level of AFP gradually returned to normal after the patient had been given anti-TB therapy for 6 months. This case was challenging because of not only the rarity of the condition (representing approximately <1% of all tubercular infections (3)) but also because there were no specific imaging findings (4). Additionally, to the best of our knowledge, portal vein thrombosis (PVT), which is associated with post-hepatitis B cirrhosis, is rarely reported in cases of hepatic TB, and the level of serum AFP was intensely elevated, which posed challenges during diagnosis. Are the non-invasive diagnostic criteria for HCC accurate when discovering a space-occupying lesion within the liver against the backdrop of hepatic cirrhosis, simultaneously with an elevated AFP level? Maybe they are not.

From this lesion, the most probable diagnosis is the lesion not showing significant enhancement in the arterial phase and showing mild to moderate delayed enhancement in the perifocal and internal septum of the venous and delayed phases. In addition, the mass presented without a capsule surrounding it and the presence of ascites and bilateral pleural effusion might suggest inflammatory fluid in this patient. Furthermore, his clinical presentation of afternoon fever and being IGRA-positive, as well as his levels of ESR and ADH being elevated, also provided a valuable diagnosis.

Nearly four-fifths (80%) of hepatic TB cases that have been reported have been owing to the systemic dissemination of tubercle bacilli or infections in the gastrointestinal tract (4). Primary hepatic TB is an extremely rare entity because the hypoxic state of the liver is not conducive for mycobacteria to grow (5–7). The pathogenesis of primary hepatic TB may be that of tubercle bacillus entering the liver from the intestine *via* the portal vein and seeding the liver parenchyma (8).

Hepatic TB most commonly ocurrs in individuals aged 11 to 50 years, yielding the peak incidence in the middle of the second decade of life. Isolated hepatic TB is more prevalent in the fourth to sixth decades of life (9). The male-to-female ratio of the disease is 2:1 (10). The clinical manifestation of primary hepatic TB is often non-specific, and most patients are minimally symptomatic or asymptomatic, whereas some patients might be associated with mild fever, weakness, weight loss, night sweats, pain in the right upper quadrant or hepatomegaly, and rarely jaundice (5, 11). Hepatomegaly is usually found with elevations of alkaline phosphatase and normal levels of aminotransferase (12).

The imaging findings of hepatic TB can be wide-ranging, but Yu et al. (13) classified it into three forms: parenchymal type (which includes macronodular and micronodular patterns), serohepatic disease, and tubercular cholangitis. This could account for the macronodular parenchymal type in this patient, with clinical presentations mimicking HCC or ICC also being reported (14). Histopathological or bacteriological confirmation is often indispensable because the imaging pattern is predominantly non-specific (9, 15–17).

A wide spectrum of imaging presentations of primary hepatic TB was reported, which could be similar to that of benign or malignant tumors. Additionally, no previously reported hepatitis cirrhosis complicated by TB has been described. However, some of the CT and MR features frequently present in HCC, such as arterial enhancement in the hepatic lesion and the subsequent washout in the portal or delayed phase (18), are unusual in cases of hepatic TB.

The ultrasonographic manifestation of hepatic TB is performed in most literature (5, 19–21), which commonly reveals solitary or multiple hypoechoic liver masses, with hyperechoic lesions seldom observed (22). This difference may be a consequence of different phases of the disease, with hyperechoic lesions representing the early stage, whereas hypoechoic lesions might represent the caseation necrosis phase.

Upon CT, classic liver tuberculoma displays hypodensity in plain imaging and often presents an unenhanced central, with barely or slightly peripheral, rim enhancement, with a central low-density lesion, owing to caseation necrosis and enhancing the peripheral rim caused by the surrounding granulation tissue. The lesions sometimes can manifest with extensive necrosis, consequently mimicking cysts, displaying no discernible peripheral enhancement (14, 23). Furthermore, various calcification patterns can be observed in CT, and the incidence of calcification ranges from 0 to 64% (24).

The magnetic resonance imaging of hepatic TB presents with a hypointense nodule or a mass with a hypointense rim on T1-weighted imaging, which is isointense, hyperintense, or hypointense with a less intense rim on T2-weighted imaging. Kale et al. reported that, in 34 out of 43 patients, dynamic contrast-enhanced MR showed internal septal enhancement or peripheral enhancement, as well as restricted diffusion (25). We encountered these features in the current case. Accordingly, MRI plays an essential role in the detection and differentiation of primary hepatic TB, and the accuracy of MRI is beyond that of CT (26).

Regarding the aspects of treatment, primary hepatic TB does not require surgery. Quadruple therapy (including the administration of ethambutol, pyrazinamide, rifampin, and isoniazid) is recommended initially for a 2-month phase, followed by 4–7 months of rifampin and isoniazid.

In summary, primary hepatic TB is rare and usually lacks typical clinical manifestations and imaging characteristics. Although no post-hepatitis B cirrhosis complicated by hepatic TB was reported, this case illustrated that the non-invasive diagnostic criteria for liver cancer should be considered carefully when discovering a space-occupying liver lesion in a hepatitis cirrhosis patient with elevated AFP. Instead, primary hepatic TB should be considered and included in the differential diagnosis, and a biopsy should be performed promptly to confirm the pathological result.

## Data availability statement

The raw data supporting the conclusions of this article will be made available by the authors, without undue reservation.

## Ethics statement

Written informed consent was obtained from the individual(s) for the publication of any potentially identifiable images or data included in this article.

## Author contributions

PL conceived the study. NH wrote the draft manuscript. MT, CL, TL, and YW collected the clinical information. SY analyzed the US imaging. YW and PL revised the manuscript. All authors contributed to the article and approved the submitted version.

## Funding

This study was partly supported by the Science and Technology Projects of Guizhou Province (Nos. Qiankehe Support [2020]4Y193 and Qiankehe Basics-ZK[2022] General 422).

## Conflict of interest

The authors declare that the research was conducted in the absence of any commercial or financial relationships that could be construed as a potential conflict of interest.

## Publisher's note

All claims expressed in this article are solely those of the authors and do not necessarily represent those of their affiliated organizations, or those of the publisher, the editors and the reviewers. Any product that may be evaluated in this article, or claim that may be made by its manufacturer, is not guaranteed or endorsed by the publisher.
